# Tutorial on Energy Transfer Mechanisms and Computational Methods in X‑ray Photodynamic Therapy with Metal Nanoclusters

**DOI:** 10.1021/acsphyschemau.5c00095

**Published:** 2026-01-01

**Authors:** Maxim Laborenz, Sami Malola, Hannu Häkkinen

**Affiliations:** † Department of Physics, Nanoscience Center, 4168 University of Jyväskylä, FI-40014 Jyväskylä, Finland; ‡ Department of Chemistry, Nanoscience Center, University of Jyväskylä, FI-40014 Jyväskylä, Finland

**Keywords:** X-ray, photodynamic therapy, X-ray photodynamic therapy, cancer, metal nanoclusters, density functional theory, gold, reactive oxygen species, energy transfer, charge transfer

## Abstract

Cancer remains the deadliest disease for mankind, and hence, the need for effective, reliable, and functioning cancer treatment is crucial. A promising minimally invasive oncological treatment called photodynamic therapy (PDT) involves irradiation of a photosensitizing drug injected into the vasculature which in turn transfers energy to the surrounding oxygen, generating heavily cytotoxic reactive oxygen species (ROS), either directly or indirectly killing the cell. Although simple in theory, many problems need to be addressed like oxygen waste and hence resupply, light source delivery to the photosensitizer (PS), or the cancer cell targeting with the PS. Promising new agents to tackle multiple issues in PDT are metal nanoclusters (NCs), especially with gold as the core. They turn out to accumulate well in cancer cells, be very biocompatible, and even function as PS themselves. A less common way to surpass the light source delivery problem is to use X-rays due to low in vivo scattering and absorption cross section, giving rise to what we will call X-ray photodynamic therapy (X-PDT). It shows great potential for demolishing cancer cells, prompted by their high energy. The energy transfer in both cases, PDT and X-PDT, from PS or NC to oxygen is poorly understood and the subject of current research. This Tutorial gives an easy to understand introduction to PDT and X-PDT and their different agents, explains the use of metal NCs in both heavily related treatment methods, gives an overview of the known elementary transfer mechanisms between the typical contributors to PDT and X-PDT, and briefly sketches realized and possible simulation strategies. It aims to give an understanding of where current research is lacking and thus what new experiments, theories, and simulations should be targeted as well as an outlook for possible further theoretical and computational X-PDT research.

## Graphical abstract

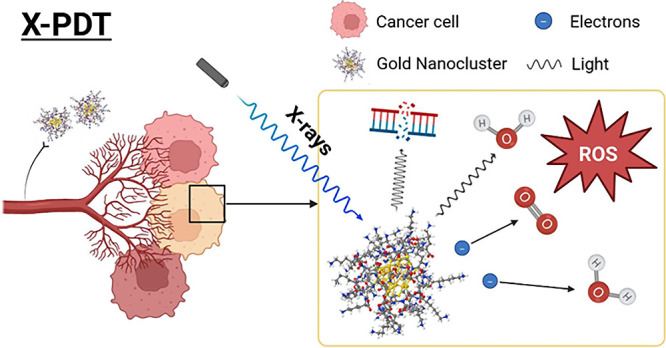

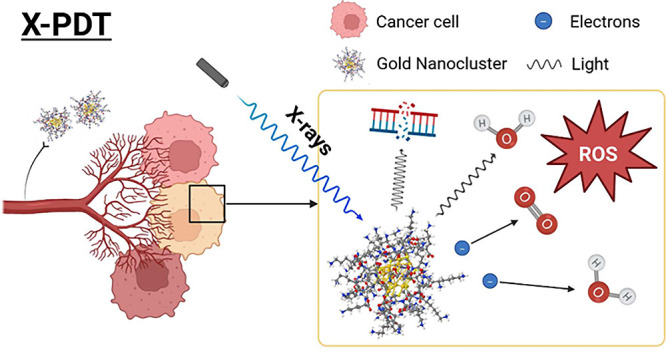

## Introduction to Photodynamic Therapy

### Motivation

A vast variety of techniques are used in modern oncology, and about 50%^1^ include ionizing irradiation to destroy DNA strands or to induce programmed cell death mechanisms and mitotic catastrophes^2^ as a first step to greatly reduce tumor size and prepare for more detailed treatment. This method, called radiotherapy (RT), has been applied with great success to many different types of cancer, like skin, prostate, lung, cervix carcinoma, or lymphomas.^3^ However, the collateral damage it causes limits its success and safety.^4–6^ In order to improve, the main issues to address are cancer targeting and source delivery to maximize efficacy and minimize side effects, especially when dealing with deeper seated tumors.

A promising candidate for this is the fairly noninvasive photodynamic therapy (PDT)^7^ in which visible or infrared light or ultrasonic waves are used to drive injected photosensitizing drugs. These in turn transfer energy and electrons to the cell environment,^8^ generating excess reactive oxygen species (ROS) like hydroxyl radicals (OH*), hydrogen peroxide (H_2_O_2_), hydroperoxyl radicals (HO_2_^*^), superoxide (O_2_^*–^), or highly reactive singlet oxygen (^1^O_2_) among others.^9,10^ In PDT, singlet oxygen ^1^O_2_ is the most important of the species in inducing cell death.^11^ It is noteworthy that there are natural reaction pathways leading to ROS as well.^12,13^ These ROS in turn can react and thus destroy the cell’s membrane, mitochondrium, or DNA strand or cause oxidative stress reactions, provoking inflammatory and immune response and leading to different possible cell death mechanisms like apoptosis, necrosis, and autophagy.^2,9^ The method in theory only needs a photosensitizer (PS), oxygen, and a light source although a new supramolecular PS agent for oxygen-independent generation of hydroxyl radicals for PDT by oxidizing water in the presence of intracellularly abundant pyruvic acid has been reported.^14^ Also, the combination of RT and PDT has been discussed excessively.^15^

### Photosensitizer and the Light Penetration Problem

Many different PS, often dyes, are in use, like temoporfin,^16^ verteporfin,^17^ or different porphyrin derivatives.^18^ Beyond dyes, e.g., thiolate–metal complexes and semiconductor quantum dots, have been proposed as potential PS as well.^19,20^ Many more interesting options occurred over the last decades due to the comparably low requirements of not being cytotoxic themselves, being photostable, being degradable, and emitting light carrying the transition energy from the singlet ground to excited state in oxygen. However, one of the major bottlenecks of PDT, the low penetration depth into the skin, is even worse when using visible light with penetration depths below millimeters.^21^ Diffusion and absorption have been studied extensively by Balland et al.^22^ Larue et al. concluded for an optical thickness μ = 1/(ϵ × *c*) = 0.33 cm consisting of the absorber concentration *c* and the molecular extinction coefficient ϵ that “it is necessary to have an average power that is multiplied substantially by about 400”, substantiating the difficulties in reaching deeper body layers.^23^ This optical thickness would, for example, correspond to pure water irradiated with a 980 nm laser.

One way to overcome this problem at least partly is the use of biphoton-absorbing components^24^ as schematically shown in Figure 1. It is a second-order nonlinear optical process where two photons are absorbed simultaneously for excitation into a higher electronic state. This state energy difference to the ground state corresponds to the sum of the energies of the two absorbed photons, bridging via a virtual state. A theoretical analysis within the semiclassical formalism of radiation–matter interaction is provided by I. Pérez-Arjona et al.^25^ These components make using infrared light feasible and, thus, enhance penetration depth indirectly. However, even red light about 620–750 nm which would be the light required to activate verteporfin only penetrates up to 3 mm into the skin.^26^

**Figure 1:**
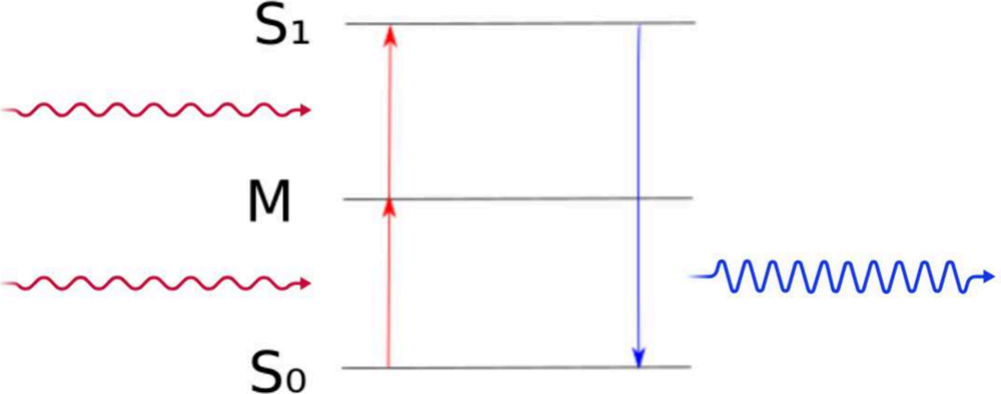
Schematic of the two-photon upconversion process, in which two photons are absorbed simultaneously. The system is brought from ground state S_0_ to an excited state S_1_ via an intermediate auxiliary energy level M as a consequence. The transition energy between S_0_ and S_1_ corresponds to the sum of both of the absorbed photons.

### Advancing PDT Performance

Another interesting improvement on PDT and RT is achieved by using metal nanoclusters (NCs).^27^ Apart from their biological advantages such as stability, efficient renal clearance, and excellent biocompatibility^28^ they also show exceptional tumor accumulation properties due to their small size and enhanced permeability and retention (EPR)^29^ as well as the nanomaterial-induced endothelial cell leakiness (NanoEL)^30^ effects demonstrated impressively by Xiao-Dong et al., who studied Au_10–12_(SG)_10–12_ biodistribution in mice.^31^ Metal NCs consist of tens to a few hundreds of atoms and contain a metal core and a shell of surrounding ligands, which can help in protecting the core and providing water solubility. Gold NCs are especially interesting in PDT applications because of their high absorbance and stability as well as their strong near-infrared excitation, ideal for ROS generation.^32^

Gold NCs consisting of ten to more than multiple hundreds of gold atoms have been successfully and reproducibly synthesized with formulas such as Au_25_SR_18_ or Au_102_SR_44_ ^33,34^ as powders or diffractable crystals. They do not just differ in size but also in their ligands and chemical composition with a significant impact on their electronic structure and thus their reactivity with a biological environment.^35,36^ Yet another advantage is that many of their structures can be determined experimentally with atomic precision using, e.g., X-ray diffraction,^37^ or studied theoretically by computational means using density functional theory based code, e.g., the GPAW package.^38,39^

As for how NCs can be used in PDT, there is an abundance of options for these versatile objects. Once again, penetration depth can be increased indirectly by using infrared light on upconverting NCs, turning it into light in the absorption regime of the injected photosensitizer for activation. Mitsui and Uchida, e.g., reported [PtAg_28_(BDT)­(12)]­(4—) (BDT = 1,3-benzenedithiolate) to enable triplet–triplet annihilation upconversion (TTA-UC) at low excitation intensities.^40^ A report on two-photon absorption cross sections for Au_10_ and Au_25_ is available.^41,42^

Another way would be to use NCs as PS themselves,^43–54^ taking advantage of the high photoabsorbance of the metal core. The NC’s high stability would allow for safe transport through the vasculature, and after accumulation in cancer cells due to EPR, their activation via light would cause meaningful harm in a targeted manner without energy losses during transfer to other photosensitizing agents. Thanks to great cellular uptake and targeting for specificity, such NCs are called third-generation photosensitizers as opposed to first-gen PS like hematoporphyrin or second-gen PS with slightly improved features such as verteporfin.^55^

In NCs, not only the core but also the ligands are involved in ROS generation beyond their functions to passivate the cluster and make it water-soluble. We simplistically understand the NC-mediated light to oxygen transfer, such that the photon absorption occurs predominantly within the delocalized electronic states of the metallic core. The excited energy is then redistributed through strong electronic coupling between the core and ligand shell, giving rise to metal-to-ligand charge transfer states. These interfacial excitations effectively channel the absorbed photon energy into the ligand framework, from which it is subsequently transferred to molecular oxygen, generating ROS. Furthermore, ligands also regulate the number of valence electrons in the metallic core^37^ and therefore crucially determine the electronic structure of the NC. As for experimental proof of ligand participation in the PDT procedure, Fakhouri et al. report their “[effect on efficiency] of gold clusters in solution to produce singlet oxygen upon excitation with visible light in a one-photon regime”. They also observed an increase in ROS above the endogenous level under one- and two-photon excitation at 473 and 720 nm for AcCys_18_, different from Au_10_ which also generated small amounts of ROS even without photoexcitation.^32^

The small ROS generation without activation through gold NCs implies permanently available low probability energy transfer channels between NCs and oxygen and thus side effects in noncancer cells as well as, e.g., found by Lillo et al. in a study on BSA-capped gold nanoclusters without irradiation.^56^ The underlying mechanisms, however, are the subject of current research.

### Improving Light Penetration

X-rays with an energy ranging from hundreds of eV to hundreds of keV undergo little absorption and scattering in vivo^55^ and consequently change to X-rays, the as light source is another method to deal with the light penetration problem, i.e., get access to deep-seated tumors. The higher energy also allows for more cytotoxic reaction pathways because the cascade of light and electrons drives a wider range of transitions and reactions. The cytotoxicity is not mainly relying on energy transfer to oxygen anymore but includes direct DNA-strand breaking and radiolysis of the surrounding water, the most abundant molecule in cells, for hydroxyl generation. X-PDT can be understood as a combination of RT and PDT, providing benefits of both treatment methods.^57,58^ In this section, we discuss two nanoplatforms capable of emitting scintillating or persistent luminescence suitable for inducing cell death: Nanoscintillators (NS) and Nanoparticles (NP).

#### Scintillators in X-PDT

Scintillators are a convenient way to ensure strong absorbance of the incident X-ray beam and efficient conversion to a desired energy via photon or electron emission,^59,60^ and propositions on how to apply them in biomedicine exist already.^61,62^ They can serve as a bridge between X-rays and the PS.

Over the last 15 years, significant progress has been made in synthesizing tailored NS to have high absorbance, improved FRET rates (an energy transfer mechanism explained in later sections), and boosted Type I ROS production, among other things that are useful for X-PDT.^63^ Reference 63 provides a comprehensive list of NS with different advantages, but there are multiple criteria that need to be met so the scintillator is useful as an X-PDT agent. Because the scintillator accumulates in the cancer cells, it needs to be on the nanometer scale to make use of EPR. We call them nanoscintillators (NS). To minimize X-ray-induced side effects, the NS conversion rate must be as high as possible, so the irradiation dose can be reduced. One way of achieving that is “codoping” the NS as demonstrated for Gd^3+^, Tb^3+^ codoped CeF_3_.^64^ Another problem to look out for is that the NS needs to be stable, of course. High-dose X-rays can cause damage and dampen scintillation^65^ or even completely destroy the scintillator.^66,67^ Stability is evaluated by comparing the quantum yield of the NS over multiple irradiation cycles, and Table 1 in ref 63 also accounts for these.

As for transportation of the NS to cancer cells, Secchi proposed nanoparticles as a mount for NS and an arrangement of PS in a radial mesh around it.^9^ She also mentions the additional requirement of a small scintillator to PS distance for all efficient energy transfers to happen. Achieving all of this would be a demanding task from an engineering point of view and is, therefore, just a design concept for now.

It is clearly not optimal to rely on both NS and PS undergoing cellular uptake in cancer cells and ending up close to each other. Luckily, there exist NS, that emit at the singlet oxygen transition and thus are the PS themselves, skipping an intermediate transfer step. Popular examples are Y_2_O_3_:Eu and NaCeF_4_:Gd.^68,69^

#### Metal Nanoclusters in X-PDT

As an alternative to NS converting X-rays to drive type 2 PDT, we can use metal NCs to deposit X-ray energy in our target cells. Since the absorbance taken from the Lambert–Beer law μ is proportional to the atomic number *Z* via $\mu ∼\frac{\rho Z^{4}}{{\mathrm{AE}}^{3}}$ (ρ being the density, *A* the atomic mass, and *E* the energy of the radiation source), heavy metal NCs can serve as excellent absorbers. The big advantage of metal NCs is their customizability.

The general idea is to customize them to be great absorbers and hence deposit an immense amount of energy in our target cells, which in turn causes a cascade of effects, driving a variety of cell kill mechanisms. On one hand, energy can be down cascaded via multiple inelastic scattering processes inside the molecule to generate photons and electrons for conventional PDT, i.e., ROS production.^70–72^ On the other hand it is possible to immediately destroy the cell by, for example, ionizing the DNA helix with Rayleigh or Compton scattered light or high energy electrons, produced during an early stage of the cascade.

Understanding and modeling the cascade is highly complex, as in addition to the relevant mechanisms for PDT outlined in later sections a lot of famous textbook mechanisms gain relevance in the X-ray regime, such as Compton electron ejection or more general “ionization”, electron–electron collision, bremsstrahlung, and Auger electron ejection. The fluorescence yield and the Auger and Coster–Kronig (a special case of the Auger effect, where the vacancy is filled by an electron from the same inner shell) transition probability depend on *Z* and have been thoroughly catalogued by Bambynek et al.^73^

It is even possible that nuclei inside a molecule become strongly ionized, which leads to a strong Coulomb repulsion between the cores mediated by their high positive charge, ultimately destroying the molecule, which is not necessarily bad as it could also potentially kill the cell, dependent on the emitted product. This mechanism is called Coulomb explosion and can already happen in the high energy X-ray regime.^74^ Experiments have already shown that, e.g., radiation damage to tumors loaded with X-ray absorbing metal microspheres at 1% by weight is increased by up to 50%^75^ and motivate further research on NC-driven X-PDT.

Both NS- and NC-driven X-PDT have the following in common: after either a scintillation process or an energy down cascade they become conventional PDT again. Thus, it is also integral for X-PDT to understand the basic effects underlying PDT, which will be discussed in the following sections.

## Energy Transfer Mechanisms between PS and ROS

### Intersystem Crossing (ISC)

Many effects in PDT lack a solid theoretical foundation, such as, e.g., the Au_10_SG_10_ ROS production without the presence of light that Fakhouri et al. claim to be “most likely attributed to the redox-active regions of the SG ligands, such as free thiol, amine, or carboxyl groups”.^32^ Not just the region but also the exchange mechanism between PS and ROS are yet to be unravelled. This section, on the other hand, summarizes the involved underlying physical mechanisms that are well understood to provide a basis for further use in theory and simulations.

Many interactions between PS and ROS or in general a donor and an acceptor molecule begin with populating an excited donor triplet state because of its longer lifetime. It is longer because changing spin multiplicity 2*S* + 1, where *S* is the total spin, is prohibited, and thus the decay rate to the donors singlet ground state *S*_0_ is greatly reduced. As a consequence, the energy transition rate to another electronic system, especially if it is dioxygen whose ground state is a triplet state, is increased. However, a sound question to ask is how a system is supposed to ever reach a triplet state when always starting at a singlet ground state if a total spin change is prohibited.

After all, breaking spin conservation is prohibited in nonrelativistic quantum electrodynamics. Nonrelativistic electrodynamics governed by the Hamiltonian *H*_K+C_ comprised of kinetic and Coulomb terms does not include a spin term, and therefore spin *Ŝ* is automatically conserved, as indicated by the vanishing commutator [*H*_K+C_, *Ŝ* ^2^] = 0.

In a fully relativistic treatment following Dirac’s famous equations, however,^76^ a spin-mixing term allowing for symmetry breaking is introduced in the form of the spin–orbit coupling (SOC) contribution. If we consider an electron with velocity *v⃗* in the external electric field generated by the nucleic charge *E⃗* it generates a magnetic field *B⃗* following Maxwell’s rules. Considering no other external magnetic fields and applying a Lorentz transformation to the relevant electromagnetic field tensor element gives a formula for the strength of the magnetic field induced by the electron

1 $$\mathrm{B⃗}=-\gamma \frac{\mathrm{v⃗}}{c}\times \mathrm{E⃗}$$

where γ is the relativistic factor $\frac{1}{\sqrt{1-{\left(v/c\right)}^{2}}}$ . If we change to the electron’s reference frame, its magnetic moment μ⃗ (related to the spin operator $\mathrm{\mu ⃗}=\frac{e}{m_{e}c}Ŝ$ ) couples to the induced magnetic field and under reexpression of the electric field via the atom’s central potential $\mathrm{E⃗}={\mathrm{E⃗}}_{r}=-\frac{d\phi \left(r\right)}{dr}\mathrm{r⃗}$ the interaction Hamiltonian becomes

2 $$H^{\prime }=-\frac{1}{2}\mathrm{\mu ⃗}\cdot \mathrm{B⃗}=f\left(r\right)\mathrm{L̂}\cdot Ŝ$$

where $f\left(r\right)=\frac{e}{2m_{e}^{2}c^{2}r}\frac{d\phi \left(r\right)}{dr}$ and *L̂* is the angular momentum operator. The product *L̂*·*Ŝ* is eponymous for SOC. This term evidently does not necessarily commute with the spin operator anymore, and therefore spin conservation is broken with the consequence of allowing for state transitions from singlet to triplet and vice versa, enabling efficient intermolecular energy transfer, in our case from PS to ROS. Such a transition between two states of different spin multiplicity is called intersystem crossing (ISC) and occurs efficiently in heavy-atom systems where even valence electrons are heavily relativistic.^77^ In practice, the computationally heavy SOC Hamiltonian is often replaced by the scalar relativistic Breit–Pauli spin–orbit Hamiltonian.^78^ Other spin couplings allowing for ISC are spin–spin and hyperfine coupling, but SOC is by far the most important one.^79^ In order to keep track of intermolecular excitation dynamics, it is useful to draw a Jablonski diagram. Such a diagram, shown in Figure 2 for an arbitrary system, visualizes all possible transitions between quantized electronic and vibrational states in a molecule, including ISC between the first singlet excited state S_1_ and the first triplet excited state T_1_.

**Figure 2:**
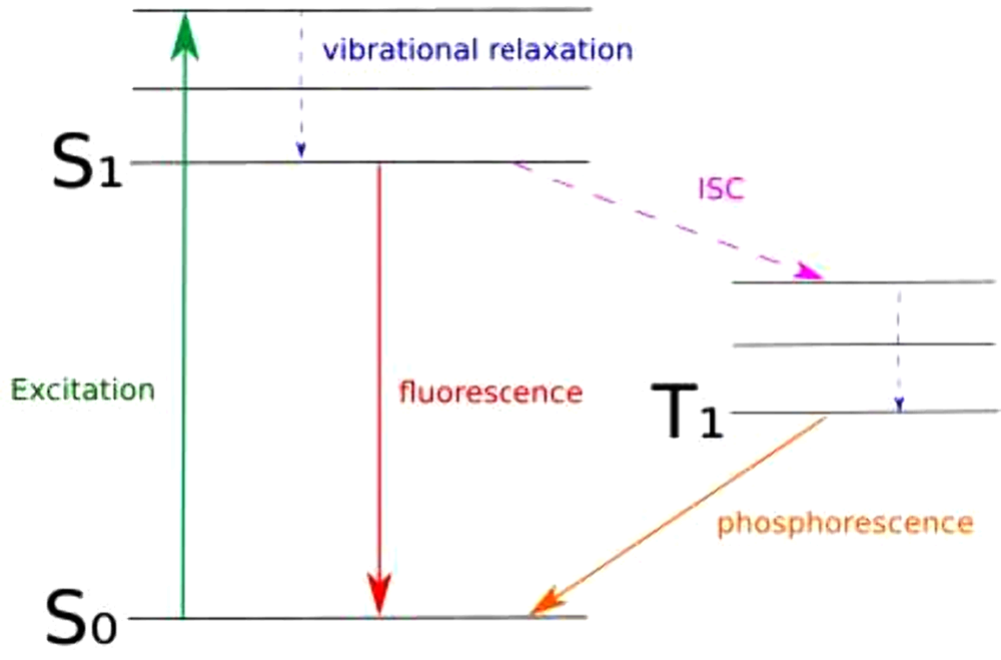
Jablonski diagram of a sketch molecule with some of the possible transition mechanisms between the different electronic states including ISC.

Another potentially relevant effect to consider in energy exchange analysis induced by SOC is its fine structure splitting of energy levels. It can lead to accidental state mixing, meaning that an energy level can be split very close to another one, enhancing transitions according to the energy gap law. It states that the rate of a nonradiative transition like, e.g., ISC decreases exponentially with increasing energy gap Δ*E* related to vibrational wave function overlap according to Franck–Condon arguments if operating in the weak coupling regime. Weak coupling regime means that the geometrical displacement of the final state minima with respect to the initial state is small.^80^ The energy gap law also appears in Fermi’s golden rule when treating electronic and vibrational levels independently by simply multiplying it by the Franck–Condon weighted density of vibrational states. However, a newer report from Penfold et al. emphasizes the importance of considering electronic and vibrational dynamics dependently for a more profound understanding of ISC beyond the Jablonski diagram and also simulated the ISC enhancement by vibrational spin–orbit coupling in porphyrin.^80^ While being computationally of potential interest for the case of dyes such as PS like porphyrin, it is not feasible yet for NCs with hundreds of atoms.

Returning to the basic SOC energy level splitting without vibrations, it scales roughly with the fourth power of the atomic number ∼*Z* ^4^, but the SOC matrix elements (SOCMEs) do not necessarily.

#### El Sayed’s Rule

The rules states that transitions between different molecular orbital types have larger SOCMEs, i.e., probability according to Fermi’s golden rule,^81^ and are not simply following the approximate *Z* ^4^ scaling. This rule successfully predicts that in some cases the SOC matrix elements between metal-to-ligand charge transfer states are smaller for gold than for copper.^80^

ISC was considered to be negligible for a long time because luminescence and internal conversion (spin-allowed radiationless transition) happen during the first few picoseconds after an excitation until it was found that ISC operates on the same time scale and is therefore competitive.^78^ Now it is a key component in understanding PDT.

However, the simulation framework for ISC is scarce, and current leading edge code packages like GPAWs LrTDDFT already need heavy approximations to yield calculations with a computational cost reasonable for current supercomputers; for example, the exact SOC is dropped and replaced by a scalar relativistic field instead. Additionally it mainly works with single excitations.^38,39^ Hence spin-vibrational quantum dynamics for clusters consisting of hundreds of atoms is currently not accessible.

### Förster- and Dexter-Type Mechanisms

After an excitation in a suited PS reaches a metastable triplet excited state, it can be transferred effectively to oxygen for ROS generation. Mechanisms responsible for such donor–acceptor transfer are categorized into two types. Type I covers electron and hydrogen atom transfer from PS to either oxygen to create superoxide O_2_^*–^, hydroxyl radicals OH*, or hydrogen peroxide H_2_O_2_, or to the biological environment.

Type II covers energy transfer pathways generating singlet oxygen ^1^ *O*_2_ for fast and localized cell destruction, and two mechanisms are usually considered in PDT. The first is the Förster resonance energy transfer (FRET) arising from dipole–dipole interaction between donor and acceptor molecules simultaneously relaxing an electron in the former and exciting one in the latter under conservation of spin in the donor and acceptor. The FRET rate *k*_FRET_ is calculated using the dipole–dipole matrix element in Fermi’s Golden Rule yielding

3 $$k_{\mathrm{FRET}}=\frac{1}{\tau_{D}}{\left(\frac{R_{0}}{r}\right)}^{6}$$

where τ_D_ is the donor fluorescence lifetime, *r* the intermolecular distance, and *R*_0_ the FRET radius which in turn depends on the normalized spectral overlap integral *J* serving as a measure of the shared energy bands available for electronic transitions:^82^

4 $$J=\int_{0}^{\infty }d\lambda f_{D}\left(\lambda \right)\epsilon_{A}\left(\lambda \right)\lambda^{4}$$

Here, *f*_D_(λ) is the donor emission spectrum and ϵ_A_ the acceptor molar extinction coefficient related to the acceptor molecule absorptivity. A larger overlap integral indicates a greater number of energetically matched states, which enhances the probability of resonant dipole–dipole interactions and facilitates more efficient FRET. FRET’s $\frac{1}{r^{6}}$ distance dependence which was experimentally proven in 1967 already^83^ leads to an estimated effective range of up to 10 nm in vacuum.

It is noteworthy that the classical FRET theory has been developed under consideration of a single donor and acceptor but has been expanded to multiple donors and acceptors already which is relevant for PDT since atoms in NCs and dyes have distances significantly smaller than the wavelength of the driving light field, giving rise to coherent and simultaneous excitation.^84^ The transfer of excitation from such a collective state modifies the single donor transition rate dependent on the specific superposition driven.

The second mechanism is the so-called Dexter transfer^85^ where the donor excitation is exchanged by swapping electrons. Its rate is given by

5 $$k_{\mathrm{Dexter}}=KJ^{\prime }e^{-2r/L}$$

where *L* is the sum of the involved molecules of van der Waals radii and *J*′ is another normalized spectral overlap integral defined as

6 $$J^{\prime }=\int_{0}^{\infty }d\lambda f_{D}\left(\lambda \right)f_{A}\left(\lambda \right)$$

where *f*_A_(λ) is the acceptor absorption spectrum. Different from FRET, Dexter exchange relies not only on spectral but also on wave function overlap of frontier orbitals (HOMO–LUMO) as well as the encoded electronic coupling constant *K*. Due to the necessity of orbital overlap, Dexter exchange is estimated to be only effective in a range up to 1 nm. Because it is a nonphotonic exchange it is also capable of transferring a triplet/singlet state in the donor to the acceptor conserving total but not individual spin in the molecules. Figure 3 visualizes how electrons are (de)­excited or exchanged during both Type II mechanisms. The arrows next to the mechanism names indicate to which state the electrons highlighted with a pink or blue bubble are moving.

**Figure 3:**
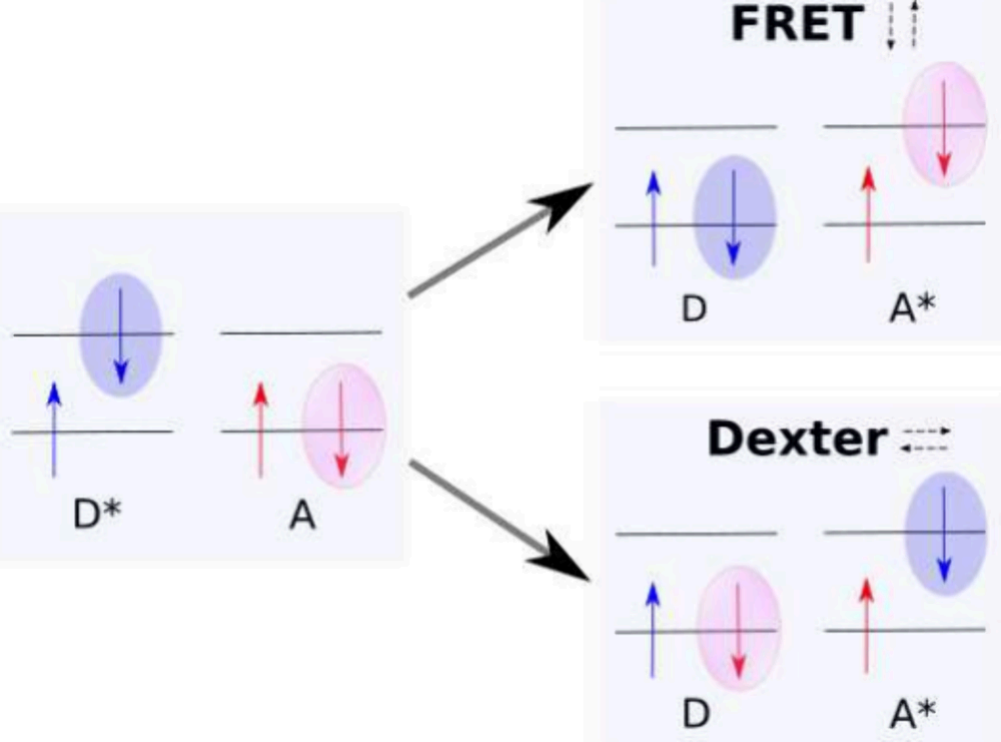
Illustration of FRET and singlet–singlet Dexter electron exchange excitation transfer.

In experiments, FRET is very useful for high precision intermolecular distance measurements as, e.g., performed by Massey et al. for various dye–DNA conjugates,^86^ but it relies on dipole–dipole interactions and is therefore only suitable for singlet–singlet or triplet–triplet transitions. Consequently a decrease in donor fluorescence yields an increase in acceptor fluorescence.

This is not necessarily the case for the Dexter mechanism. Although it can also appear in singlet–singlet or triplet–triplet scenarios, it will be mostly dominated by FRET and is therefore primarily relevant in ISC scenarios transforming a (^*n*^D*, ^*m*^A) into a (^*m*^D, ^*n*^A*) pair (*n* ≠ *m*). This allows for a decrease in donor fluorescence after ISC without an increase in acceptor fluorescence because of the transfer of a triplet state causing low acceptor quantum yield since *T*_1_ → *S*_0_ is spin forbidden. The most famous Dexter mechanism example is triplet–triplet annihilation (TTA)^87,88^ where two excited triplet state molecules transform into one molecule in the singlet ground and the other in the singlet excited state ^3^D* + ^3^A* → ^1^D + ^1^A**. The nature of TTA implies upconversion. Dexter exchange-type mechanisms will also be dominant when encountering molecular compounds with little spectral overlap since FRET heavily depends on it.^89^

### Charge Transfer

Converting oxygen to ROS via charge transfer (CT), i.e., transfer of an electron or hole such that D + A → D^±^ + A^∓^, is a reaction pathway for Type I PDT. For example, electron transfer to oxygen can generate the cytotoxic superanion species *O*_2_^*–^. By now there is a plethora of CT theories, and most of them consider it to be an excited state effect. This section only covers the most influential one focusing on electron transfer (ET) in the solution phase as relevant to PDT due to the aqueous environment inside cells and scratches pivotal extensions to it.

Three distinctions can be made for CT. The first is differentiating between a direct CT without an intermediate step and a unistep superexchange mediated process D* encoded BA → D^+^BA^–^ or a multistep sequential process D*BA → D^+^B^–^A → D^+^BA– transferring charge in materials, particularly in DNA between two sites through a mediating atom or molecule depicted as B. Such an indirect transfer allows for longer range charge transfer up to 300 Å.^90–95^

Second, a distinction between *outer sphere CT* and *inner sphere CT* is made. In outer sphere CT the donor and acceptor retain identities; i.e., no bonds are formed or broken, and electron transfer occurs via solvent-mediated interactions. In inner sphere CT the reactants’ geometry is distorted and can even involve actual changes in the chemical species in the product state.

The third distinction is between adiabatic and diabatic or nonadiabatic CT (a helpful visualization is provided in Figure 4).

**Figure 4:**
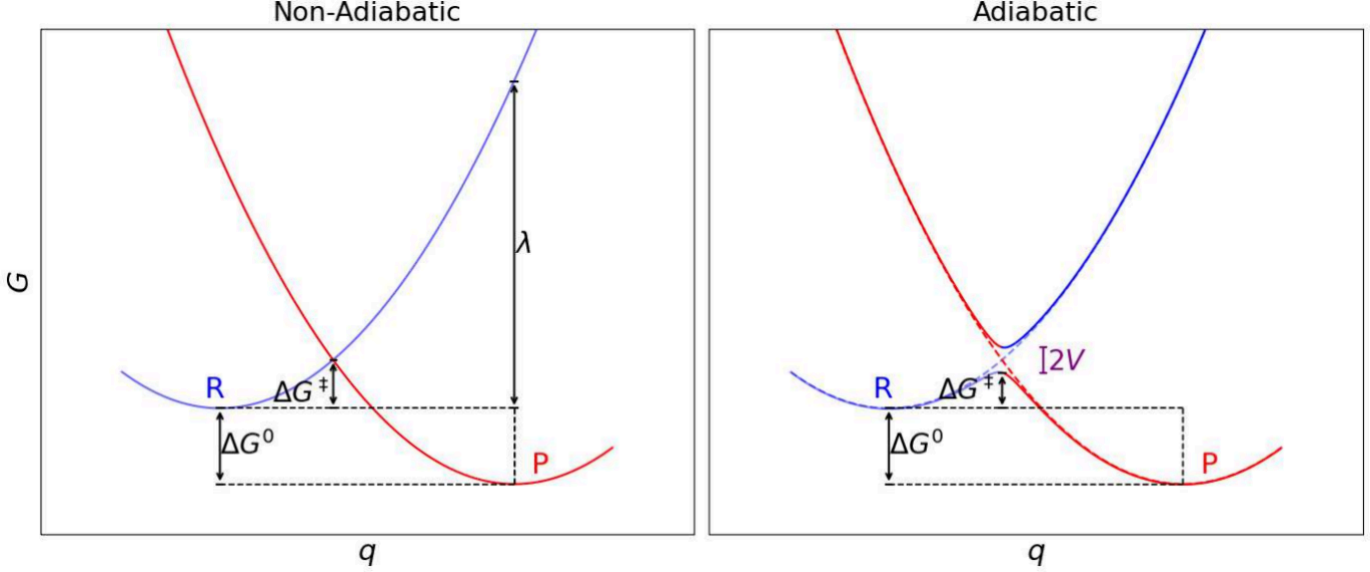
Sketch of arbitrary Marcus parabolas and relevant quantities necessary for their displacement construction such as the activation free energy Δ*G* ^‡^. Blue denotes the reactant and red the product state free energy. Electron transfer occurs at the intersection of both parabolas.

For a sophisticated description of CT vibrational modes, they have to be included. A first extension for the rate expression provided by Fermi’s golden rule is to multiply it with the Franck–Condon factor *F* such that

7 $$k=\frac{2\pi }{\hbar }|V{|}^{2}F$$

with |*V*| being the electronic coupling matrix element.^96^ The goal of CT theory must be to find an expression for *F*.

#### Marcus Theory

A popular semiclassical description of ET between reactants (in solution) awarded with a Nobel prize in 1992 is Marcus theory. Here ET depends on the change in Gibbs free energy Δ*G*(*p*,*T*), which is the thermodynamic potential minimized when a system reaches chemical equilibrium at constant pressure *p* and temperature *T*, due to nuclear reorganization when moving from the reactant to the product state. It also indicates if a transfer can happen spontaneously or not, depending on the sign. If Δ*G* is negative, the process can happen spontaneously.

Marcus theory can be conveniently visualized plotting the free energy of the reactant and the product state against a reaction coordinate, e.g., the solvent polarization or bond length. The free energy in basic theory will form a parabola under the assumption that the reorganization of nuclei behaves like a harmonic oscillator. The product state free energy is then shifted in energy by the negative reaction free/free energy difference −Δ*G* ^0^ and displaced along the reaction coordinate such that the sum of reorganization energy λ and Δ*G* ^0^ taken from the minimum of the product parabola intersects the reactant parabola. The overlapping parabolas then form an energy surface. Figure 4 shows a sketch of such a Marcus parabola construction.

In the nonadiabatic case treated in Marcus original theory,^97^ ET happens as hopping at the intersection of both parabolas which applies in the weak electronic coupling regime. This means that an activation free energy Δ*G* ^‡^ is required for the transfer to occur, given by Marcus’ famous equation

8 $$\Delta G^{‡}=\frac{{\left(\lambda +\Delta G^{0}\right)}^{2}}{4\lambda }$$

From Fermi’s golden rule for these nonadiabatic donor and acceptor states in the weak coupling regime case, the ET rate^98^ can be deduced to be

9 $$k_{\mathrm{na}}=\frac{2\pi }{\hbar }|V{|}^{2}\frac{1}{\sqrt{4\pi \lambda k_{B}T}}e^{-\Delta G^{‡}/k_{B}T}$$

In the adiabatic/strong coupling case the parabola intersection region is replaced by a gap of size 2*V*, and the electron transfer is accompanied by a smooth change in free energy following the energy surface from reactant to product state minimum.

A helpful tool to distinguish between the weak and strong coupling regime related to (non)­adiabaticity is the Landau–Zener parameter $\gamma =\frac{2V^{2}}{\hbar \omega_{m}\sqrt{\lambda_{m}\hbar \omega_{m}}}$ where λ_*m*_ and ω_*m*_ are the reorganization energy and effective medium frequency of vibrational mode *m*. γ < 1 indicates the weak and γ > 1 the strong coupling regime.

Just to briefly mention what to search for when interested in the adiabatic case, i.e., γ > 1, the Holstein model for interaction between phonons and electrons predicts the CT rate between two molecular sites to be

10 $$k_{\mathrm{ad}}=\frac{\omega_{m}}{2\pi }e^{-\Delta G^{‡}/k_{B}T}$$

where ω_*m*_ denotes an effective medium frequency of mode *m*.^99^

Although very successful, limitations of Marcus theory are at hand, for example, the operation in the nonadiabatic weak coupling regime along a single reaction coordinate while treating nuclei as classical and the states as harmonic oscillators.

#### Marcus–Levich–Jortner Theory

A robust extension of the classical theory including quantized nuclear vibrations into the calculation of the ET rate is captured in the Marcus–Levich–Jortner (MLJ) theory^100,101^ in which the system is divided into a quantum mechanical subsystem including the reactants and a classical solvent bath. MLJ theory is of particular interest in studies of molecular ET reactions which are strongly exothermic^102^ or initiated by photoexcitation.^103,104^ It is also particularly useful in the inverted region, where the thermal driving of strength ϵ is larger than the reorganization energy. Tunneling becomes significant then,^96^ yielding a lower ET rate given by a sum over vibrational states of the initial and the final state μ and ν

11 $$k_{\mathrm{MLJ}}\left(\epsilon \right)=\underset{\mu \nu }{\sum }\frac{\Delta^{2}}{\hbar }\sqrt{\frac{\pi \beta }{\Lambda^{b}}}\frac{e^{-\beta E_{R}^{\mu }}}{Z_{0}^{S}}|\theta_{\mu \nu }{|}^{2}\cdot e^{-\beta /4\Lambda^{b}\left(\Lambda^{b}-\epsilon +E_{P}^{\nu }-E_{R}^{\mu }\right)}$$

*Z*_0_^*S*^ is the reactant partition function in the energy eigen basis; Λ^*b*^ is the bath reorganization energy; Δ is the nonadiabatic coupling; *E*_*R*/*P*_^μ/ν^ is the eigen energy corresponding to the reactant product state with vibrational quantum number μ and ν, respectively, also called internal energy levels; and θ_*μν*_ is the Franck–Condon factor, i.e., vibrational overlap.

The MLJ rate expression is commonly formulated either via a semiclassical Franck–Condon sum over the vibrational states or via a cumulant expansion of the energy gap correlation function in the latter part of eq 11. More recently Heller and Richardson expressed the MLJ rate within an instanton theory framework based on Feynman path integrals^105,106^ by mapping known results from the spin-boson model to the ET problem. They reported that it turned out to be significantly more accurate than the cumulant expansion or the semiclassical Franck–Condon sum and additionally obeys detailed balance,^107^ i.e., that at equilibrium the rates of direct and reverse processes must be equal.

#### Conical Intersections

These theories are only the tip of the iceberg, and many more mechanisms, models, and extensions have been developed. For example, decent progress has been made by experimentally and theoretically investigating the role of specific intersection topographies.^108,109^ It turns out that specific topographies lead to specific reactive outcomes for trajectories passing through the region of strong nonadiabatic coupling with the so-called conical intersections (CIs) as noteworthy examples due to their frequent appearance in experiments.^110–113^ CIs are now generally accepted as being the dominant source of coupled charge and vibrational energy flow in the molecular excited states. In the quantum chemical framework for CI, different from Marcus harmonic parabolas, ab initio calculatable potential energy surfaces (PES) of the reactant and product state are in contact at a point in nuclear configuration space. According to Schuurman and Stolow, the descriptive “coordinate space in which the degeneracy is lifted linearly at a point of intersection between two nonrelativistic electronic states” is two-dimensional and is termed *branching space*.^114^ The *N* – 2 other degrees of freedom define the so-called *seam space*. The branching space can be constructed using only intuitive quantities, namely, the reactant and product state vectors |Ψ_*R*/*P*_⟩, dependent on electronic and nuclear coordinates and the system’s electronic Hamiltonian *H* via the energy difference gradient **g** and the nonadiabatic coupling vectors **h**.

12 $$\begin{array}{l} g=\frac{1}{2}\left(\langle \Psi_{R}|\nabla H\left|\Psi_{R}\right\rangle -\langle \Psi_{P}|\nabla H\left|\Psi_{P}\right\rangle \right)\\ h=\left\langle \Psi_{R}\right|\nabla H\left|\Psi_{P}\right\rangle \end{array}$$

Now, rotating **g** and **h** to be orthogonal together with the seam space forms a set of intersection-adapted coordinates.

Contact points between these PES, i.e., CI, allow for ultrafast transitions. Examples where CI can be applied are photoisomerization,^112^ internal conversion,^115^ and photochemical reactions.^116,117^

### Plasmon-Enhanced PDT

Plasmons are quantized oscillations of a collective free electron gas as approximately found in metals, doped semiconductors, or as in our case metal–ligand interfaces where they appear as so-called surface plasmons because their net effect of oscillations is seen at surfaces or interfaces. In the classical Drude model for a free electron gas the plasmon frequency ω_*p*_ is found to only depend on the electron density *n* via

13 $$\omega_{p}=\sqrt{\frac{ne^{2}}{\epsilon_{0}m}}$$

where *e* and *m* are electron charge and mass and ϵ_0_ is the dielectric permittivity. In the random phase approximation using quantum many-body theory, plasmon frequencies and momenta are found as tuples leading to zeros of the Lindhard dielectric function ϵ­(ω, *q*) which is dependent on energy ω and momentum *q* of the external perturbating field that the material ϵ­(ω, *q*) describes is responding to.^118–120^ For the optical limit, i.e., *q* → 0, the Drude plasmon frequency is recovered. Plasmons are useful for energy exchange between systems because they can either transfer energy directly to an acceptor, which is then called “plasmon-induced resonant energy transfer (PIRET)”, or decay and redistribute the energy to a single electron in the valence band. This electron is then called “hot” because it possesses energy far above the Fermi level and thus requires lower activation energy for chemical reactions.

PIRET and hot electron transfer mediated via plasmonic NPs can play a significant role in PDT. Pu and Pons, e.g., reported efficient hydroxyl production when using gold nanorods with TiO_2_ deposited at their extremities under near-infrared irradiation. They attribute this to hot electron transfer from the nanorod to the TiO_2_ tips followed by a reduction of dioxygen.^121^ Other groups report enhancement of the near-infrared emission of a PS for two-photon absorption singlet oxygen generation, mediated by plasmonic energy transfer from gold nanorods.^122^ Gold NPs are especially interesting for plasmon-enhanced PDT because of their strong plasmon absorption in the near-infrared region, and a big advantage in using plasmonic nanostructures is the surface plasmons strong dependence on morphology, size, and composition, which, in theory, gives the ability to control their energy.^123^

### Oxygen Dosimetry and Oxygen-Independent PDT

Logically a longer lifetime of the singlet oxygen state τ­(^1^ *O*_2_) is desirable as it increases the probability of it undergoing a cell death inducing reaction. It turns out that τ­(^1^ *O*_2_) can vary strongly depending on the experimental setup. Changing ligands or use of certain doping in a NC was shown to be efficient at quenching singlet oxygen. For example, using the Au_25_(SC_2_Ph)_18_ NC as PS yielded longer τ­(^1^ *O*_2_) than Au_25_(SC_3_)_18_ or Au_25_(SC_4_)_18_. Likewise, doping it with cadmium yielded improved τ­(^1^ *O*_2_) compared with doping with mercury or no doping at all. Both effects are attributed to a more positive oxidation potential of the molecular cluster which apparently corresponds to longer singlet oxygen lifetime and also enhanced photosensitization.^124^

However, the singlet oxygen pathway for PDT has the big disadvantage of depending strongly on the oxygen concentration, which poses difficulties in oxygen poor environments or when oxygen has been considerably consumed or depleted since singlet oxygen reacts with nucleic acids, lipids, and proteins^125,126^ Wang et al. qualitatively described this, deriving rate equations for the different PS and oxygen states^127^ as plotted in Figure 5. Such a macroscopic rate equation model allows for computationally cheap coupling of PS and oxygen yielding results for, e.g., singlet oxygen creation and triplet oxygen consumption for a specific irradiation induced energy deposition. It is well suited for quantifying issues related to a low oxygen concentration and estimating necessary oxygen resupply rates for efficient PDT.

**Figure 5:**
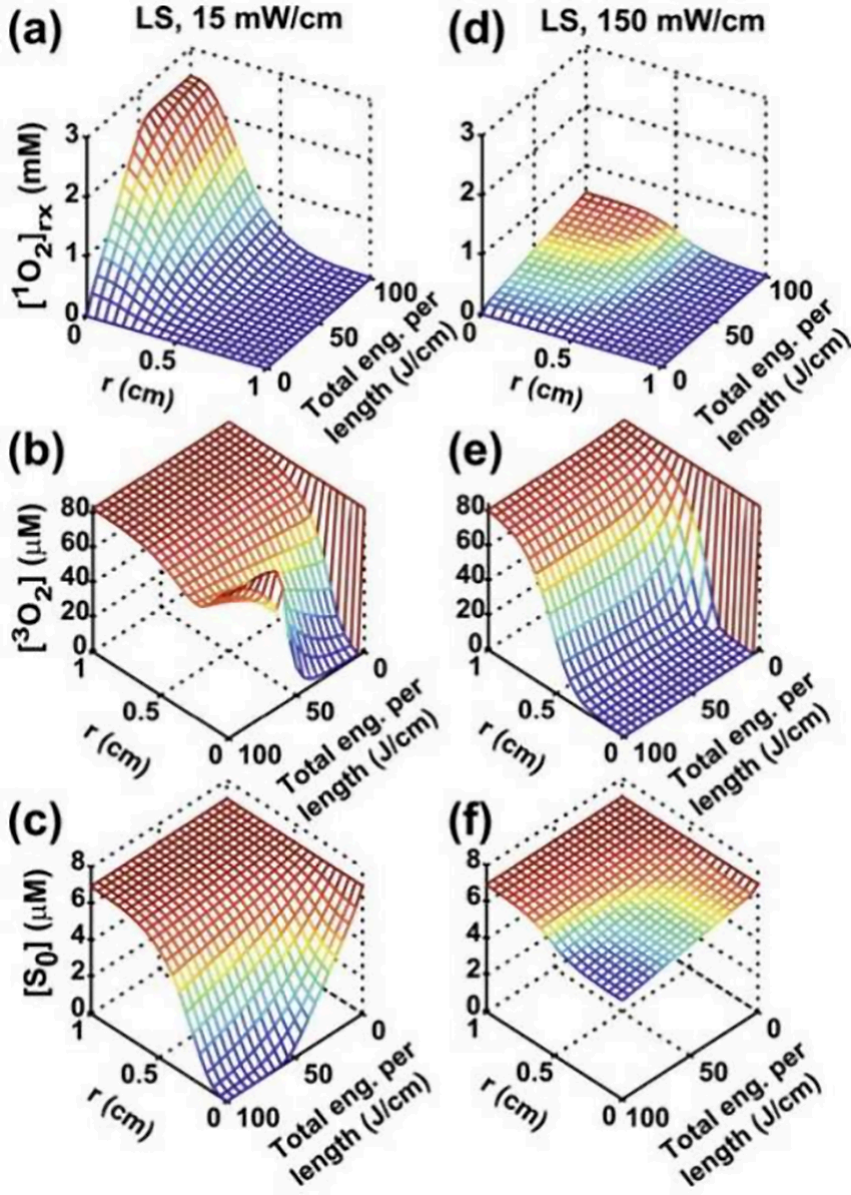
Spatially and temporally resolved distributions for (a,d) singlet oxygen [^1^ *O*_2_], (b,e) triplet [^3^ *O*_2_], and (c,f) ground state sensitizer [*S*_0_] concentration under irradiation. *r* = 0 is the center of the linear light source. Adapted with permission from ref 127. Copyright 2025 *Journal of Biophotonics*.

However, it is also possible to mitigate the resupply issue, especially tricky when dealing with tumor hypoxia, i.e., insufficient oxygen supply due to abnormal blood vessel formation, by using oxygen-independent PDT methodologies.^49^

Finding agents for oxygen-independent PDT is currently a hot topic, and new solutions are published on a monthly basis. To name some examples, coassembly of fluorene-substituted BODIPY with perylene diimide in the presence of pyruvic acid oxidizes water, generating cytotoxic hydroxyl ·*OH*.^128^ Other groups report the use of polymer-based organic PS designed to generate ·*O*_2_^–^ and ·*OH* via photoactivation of water^129^ or the combination of a conventional PS for oxygen-dependent PDT and alkoxyamin generating radicals upon irradiation, although the radicals were only detected using electron paramagnetic resonance spectroscopy and were not further specified.^130^

Oxygen independent PDT can also be enhanced by plasmonic energy transfer as Liu et al. showed an energy transfer from gold NP plasmons to Cu_2_O inside the same nanocomposite, efficiently producing singlet oxygen without the necessity for environmental oxygen.^131^

Last but not least, NC-driven X-PDT partly overcomes hypoxia due to access to radiotherapeutical killing mechanisms on top of ROS production.

## Modeling X-ray Driven Photodynamic Therapy

As mentioned before, PDT using X-rays is beneficial for deep tissue cancer treatment due to low in vivo scattering, thus circumventing the penetration problem of visible light. However, the amount of effects, contributing elements, and cascadal nature of energy deposition make X-PDT simulation and modeling a challenging task. A simulation including all factors is far out of reach, and current models focus on different aspects of this highly intertwined and complex field, often using approximated rate equations as a basis for a ROS generation estimate.

Morgan et al. (2009), e.g., estimated ROS production this way assuming the entire X-ray energy would be converted to the medium by NSs.^132^ However, Bulin et al. (2015) showed that most electrons are migrating way beyond the size of the NS.^133^ This not only means that a fraction of the deposited energy ultimately ends up being converted in the NS for singlet oxygen driving but also stresses the crucial role of the surrounding water, which will absorb most of the energy. Klein et al. (2019) decided to model the spatial energy deposition less and calculated the luminescence yield of NPs as a function of initial radiation dose, NP concentration, and scintillator light yield instead using a simplified electron cross section for NPs and tissue in aqueous surrounding. A more recent simulation by Hossein et al. (2025) from a more biological point of view consists of multiple steps modeling first tumor growth and the accompanying oxygen consumption and nutrient diffusion, then the singlet oxygen production based on the available oxygen concentration and finally estimates cell viability.^134^

Simulations of the energy deposition step focusing on individual electron generation are also available. Carter et al. (2007) counted the electrons generated by Photo and Compton effect in water using the Klein–Nishina cross section and the electrons by Photo and Auger effect in gold. The fraction of interacting electrons with gold for the generation of secondary electrons was then calculated using a modified Bethe formula while all electrons were attenuated by water, causing hydroxyl radical generation with a constant rate.^135^

Now we introduce the reader to two promising and still actively developed packages with potential in modeling different aspects of X-PDT, which are backed by extensive documentaries.

### Introduction to Geant4

Geant4-DNA^136^ is a low-energy track-structure approach extension of the open access macroscopic general-purpose radiation transport Monte Carlo (MC) C++ code Geant4. MC is considered the gold standard in clinical dosimetry in the X-ray regime and is generally well suited for simulating energy deposition in tissue. A wide range of effects is considered for the modeling of the electron cascade including elastic electron scattering, electronic excitation, ionization, and plasmonic excitation to provide highly detailed simulations. Geant4-DNA is specifically developed for DNA liquid water environments and allows for a quantitative prediction of early DNA damage including single- and double-strand break on DNA. It operates in the trajectory approximation, i.e., bulk gold in bulk water, and is therefore not suited for PDT relevant metal NCs as angular differential and integral atomic cross sections are missing. In the Geant4_DNA_Au extension these quantities are calculated via ELSEPA^137^ treating the individual atoms relativistically by solving the full Dirac equation, which is not feasible for gold NCs with hundreds of atoms on current supercomputers at all.

Geant4-DNA’s main limitation, however, is its low resolution in the low-energy irradiation regime. Sakata et al. stressed the importance of and implemented discrete electron transport models for gold including the full de-excitation cascade down to 10 eV^138^ which is still significantly beyond the singlet oxygen transition energy from the excited singlet state to the triplet ground state of about 0.98 eV.^139^

While not ideal for NC oxygen transfer modeling, Geant4 provides a framework for scintillation simulations supported by the Scintillator Simulation Library for Geant4 (**SSLG4**).^140^ The library gives access to a rich repository of scintillators for simplified scintillator handling and can be extended indefinitely. Using the Geant4 constructor of the ScintillatorBuilder class or by inheriting from the VMaterialBuilder class, it is possible to create objects for promising metal–NC scintillator candidates in X-PDT, which can be implemented in the SSLG4 for further easy to use open access scintillating metal-NC-based track structure simulations. Knowing the light yield at the singlet oxygen absorption level or the infrared for two-photon absorption of an NC from experiments could be used to derive singlet oxygen production rates for a specified oxygen density in a cell.

### Introduction to GPAW

GPAW is a multipurpose open access python based software for general-purpose density functional theory (DFT) calculations, using the projector augmented wave method framework.^39^ It allows for calculations on the atomic scale and is most commonly used for structural optimization and characterization. GPAW is well suited for ab initio calculation of absorption/emission spectra with time-dependent density functional theory or the core-hole approximation, band structure, density of states, plasmon frequencies accessed via the GW approximation, and much more.

DFT and especially time-dependent DFT (TDDFT) calculations, however, become computationally extremely heavy very quickly, for which reason many simplifications must be made. For instance, continuum states are represented by just calculating a finite number of unoccupied Kohn–Sham states above the valence band in all excited state calculations, be it TDDFT, Lr-TDDFT or the XAS module. Furthermore, GPAW mainly operates in the single particle and frozen core approximation. It is possible to make custom setups with more available bands, but in the case of heavy atoms like gold, the core must partially remain frozen due to computational demand.

Both are powerful tools for simulating X-ray interactions with matter but can independently only simulate parts of a full X-PDT procedure.

## Outlook

The bridge between macro- and microscopic models in (X-)­PDT has yet to be constructed to make use of the many classical ROS rate equation derivations reported up to now.^127,141,142^ While awaiting stronger computational resources for higher resolution track simulations and access to more complete cellular environments with more and more atoms, there are a great deal of options already for unraveling more parts concerning (X-)­PDT. We wish to close this tutorial with final notes on three possible future tasks in the GPAW framework.

First, one could use the GW approximation for quasiparticle spectra calculations.^143^ If these reveal large plasmonic bands in the near-infrared region, the NC can be relevant for PDT utilizing upconversion.

Second, one could analyze nuclear dynamics by using GPAWs Ehrenfest dynamics.^144^ In this mean field approach, nuclear movement is calculated with the velocity-verlet algorithm fed with average forces obtained from TDDFT. With the Ehrenfest approach, it is possible to obtain time-resolved ionic kinetic energies and nuclear dynamics, relevant for ROS production and cell damage analysis. TDDFT molecular dynamics (MD) calculations on compound systems of NCs and explicit dioxygen or water might give hints for how energy transfer is happening between the components when applying a continuous laser light field or when mimicking collisions between these. Furthermore, it is possible to do NC dissociation dynamics simulations under strong light fields where the size distribution over time and interatomic distances can be tracked for up to hundreds of femtoseconds. Even picoseconds might be possible when using the LCAO basis as recommended by Zobač et al.^145^

Third, one could investigate charge transfer channels through white line analysis and probing for oxidation states with site and orbital specific X-ray absorption spectroscopy (XAS).^146^ By running an MD simulation and doing XAS on the NC at different snapshots, it is also possible to thermally resolve the spectra to understand the impact of temperature on NC to oxygen energy transfer. Just recently, Johnsen et al.^147^ published a paper on their new extension to the GPAWs XAS module. In addition to K-edge XAS, L- and M-edge XAS/XES in the single-particle picture have been merged into the main master branch of GPAW. L-edge XAS is particularly valuable for gold, as the dipole-allowed 2p → 5d transitions provide direct access to the 5d valence band, which plays a crucial role in the optical properties of gold.

As a highly potent and promising treatment in oncology, any research on (X-)­PDT is valuable, and for this, a solid theoretical foundation is mandatory. We are always waiting for more thorough theoretical frameworks, their implementation, and faster supercomputers. However, as exciting as the new methodologies and the stronger hardware are, our current capabilities are not yet fully realized. It might be worth looking into already implemented modules to yield field-forwarding results. Geant4 for energy deposition and scintillation and GPAW for more detailed energy transfer mechanisms are huge open source packages whose potential is not yet exhausted, and we can expect many more field enriching papers to appear based on their current state in the future.
